# Genome-Wide Extrachromosomal Circular DNA Profiling of Paired Hepatocellular Carcinoma and Adjacent Liver Tissues

**DOI:** 10.3390/cancers15225309

**Published:** 2023-11-07

**Authors:** Jianyu Ye, Peixin Huang, Kewei Ma, Zixin Zhao, Ting Hua, Wenjing Zai, Jieliang Chen, Xiutao Fu

**Affiliations:** 1 Key Laboratory of Medical Molecular Virology (MOE & NHC), Research Unit of Cure of Chronic Hepatitis B Virus Infection (CAMS), School of Basic Medical Sciences, Shanghai Medical College Fudan University, Shanghai 200032, China; 14301010010@fudan.edu.cn (J.Y.); 19301050244@fudan.edu.cn (K.M.); 20301050296@fudan.edu.cn (Z.Z.); 16301010026@fudan.edu.cn (T.H.); zaiwjing@163.com (W.Z.); 2 Liver Cancer Institute, Fudan University, Shanghai 200032, China; huang.peixin@zs-hospital.sh.cn; 3 Department of Hepatic Oncology, Zhongshan Hospital, Fudan University, Shanghai 200032, China; 4 Department of Liver Surgery and Transplantation, Zhongshan Hospital, Fudan University, Shanghai 200032, China; 5 Key Laboratory of Carcinogenesis and Cancer Invasion (Ministry of Education), Shanghai 200032, China

**Keywords:** eccDNA, HCC, Circle-seq, chromosome 22, BAIAP2L2

## Abstract

**Simple Summary:**

Hepatocellular carcinoma is a severe cancer with various underlying causes. Extrachromosomal circular DNA, first identified in the 1960s, has gained significant attention in recent years with the development of sequencing techniques, revealing its presence in various cancer types. However, the distribution and significance of extrachromosomal circular DNA in hepatocellular carcinoma remain poorly understood. In this study, we collected eight pairs of hepatocellular carcinoma and adjacent non-tumor tissue samples, and conducted a comprehensive analysis of extrachromosomal circular DNA profiles. The results provide evidence of the extrachromosomal circular DNA expression patterns and their correlation with transcriptome dysregulation in hepatocellular carcinoma.

**Abstract:**

Hepatocellular carcinoma (HCC) develops through multiple mechanisms. While recent studies have shown the presence of extrachromosomal circular DNA (eccDNA) in most cancer types, the eccDNA expression pattern and its association with HCC remain obscure. We aimed to investigate this problem. The genome-wide eccDNA profiles of eight paired HCC and adjacent non-tumor tissue samples were comprehensively elucidated based on Circle-seq, and they were further cross-analyzed with the RNA sequencing data to determine the association between eccDNA expression and transcriptome dysregulation. A total of 60,423 unique eccDNA types were identified. Most of the detected eccDNAs were smaller than 1 kb, with a length up to 182,363 bp and a mean sizes of 674 bp (non-tumor) and 813 bp (tumor), showing a greater association with gene-rich rather than with gene-poor regions. Although there was no statistical difference in length and chromosome distribution, the eccDNA patterns between HCC and adjacent non-tumor tissues showed significant differences at both the chromosomal and single gene levels. Five of the eight HCC tissues showed significantly higher amounts of chromosome 22-derived eccDNA expression compared to the non-tumor tissue. Furthermore, two genes, SLC16A3 and BAIAP2L2, with a higher transcription level in tumor tissues, were related to eccDNAs exclusively detected in three HCC samples and were negatively associated with survival rates in HCC cohorts from public databases. These results indicate the existence and massive heterogeneity of eccDNAs in HCC and adjacent liver tissues, and suggest their potential association with dysregulated gene expression.

## 1. Introductory Statement

Liver cancer remains a global health challenge, with an estimated incidence of >1 million cases by 2025. Hepatocellular carcinoma (HCC) accounts for approximately 90% of all primary liver malignancies and typically develops in the context of various risk factors, including viral hepatitis, non-viral hepatitis, chronic alcohol consumption, autoimmune diseases, and others. The pathogenesis of HCC involves multiple molecular mechanisms, including genetic mutations, epigenetic modifications, disrupted signaling pathways, chronic inflammation, and metabolic alterations [1,2]. Globally, HCC was the sixth most diagnosed cancer and the third leading cause of cancer-related death in 2020, with approximately 906,000 new cases and 830,000 deaths [3]. Despite the development of many therapeutic treatments, overall survival when the disease is already locally advanced or metastatic is <10% in 5 years [4]. These challenges give rise to the urgent need to investigate the molecular mechanism of HCC development and to provide new insights into prognosis and treatment.

Extrachromosomal circular DNA (eccDNA) was first identified by Alix Bassel while investigating the theory suggested by Franklin Stahl in 1964 that chromosomes of higher organisms are made of a series of DNA circles [5]. Recent studies have shown that eccDNA is more prevalent in human tissues than previously anticipated [6]. Based on length, eccDNA can be divided into at least two classes as follows, small eccDNAs and large extrachromosomal DNAs (ecDNAs). EccDNAs range from several hundred to several thousand base pairs (bps) with relatively high copy numbers. EcDNAs can extend to as long as several million base pairs and typically carry oncogenes or dru-resistance genes [7,8]. Both eccDNAs and ecDNAs have massive heterogeneity in length, copy number, and origin in various tissues and cell lines. The presence of eccDNA is linked to normal physiology functions and certain phenotypes, and it may be involved in genome plasticity and evolution [9]. In addition, eccDNA may also produce small regulatory RNAs to regulate gene expression in a promoter-independent manner [10]. Although tens of thousands of eccDNAs have been identified by pan-cancer analysis of various tumor types [11], the previous analysis mainly focused on tumor cell lines or tissue samples, and it lacked comparison of the distribution pattern of eccDNAs in HCC tissue and matched non-tumor liver tissue.

Here, we comprehensively investigated the differential eccDNA profile of eight matched HCC and adjacent non-tumor tissue samples. Although the overall length and chromosome distribution of eccDNAs were similar between the HCC tissue and adjacent non-tumor tissue, the eccDNA expression in the HCC tissue showed some characteristics at the chromosome and gene level. Five of the eight HCC tissues showed significantly higher amounts of chromosome 22 (chr22)—derived eccDNA expression compared to the non-tumor tissue. Two genes, SLC16A3 and BAIAP2L2, which exhibited higher expression levels in HCC tissues, were related to eccDNA types exclusively detected in HCC samples and showed an association with lower survival rates in independent HCC patient cohorts.

## 2. Materials and Methods

### 2.1. Patients and Tissues

We recruited eight HCC patients who underwent radical liver resection and were pathologically diagnosed with solitary HCC. There was no cancerous tissue involved in the liver resection margins. Detailed clinical characteristics of patients are shown in Supplementary Table S1. The Ethics Committee of Zhongshan Hospital approved the study design (No. Y2021-125), and written informed consent was obtained from the patients. All the adjacent non-tumor tissues we obtained for analysis underwent imaging, visual differentiation, and pathology confirmation to ensure their distinction from the HCC tissue [12].

### 2.2. Tissue DNA Preparation and eccDNA Sequencing

High-throughput eccDNA sequencing was performed by CloudSeq Biotech Inc. (Shanghai, China). Matched tumor and non-tumor liver tissues were suspended in L1 solution (Plasmid Mini AX; A&A Biotechnology, Pomorskie Gdynia, Poland) supplemented with Proteinase K (ThermoFisher, Waltham, MA, USA) and incubated overnight at 50 °C with agitation. After lysis, samples were treated with alkaline, followed by precipitation of proteins and separation of chromosomal DNA from circular DNA through an ion exchange membrane column (Plasmid Mini AX; A&A Biotechnology). The linear DNA was removed by exonuclease treatment (Plasmid-Safe ATP-dependent DNase, Epicentre, Madison, WI, USA) at 37 °C in a heating block and the enzyme reaction was carried out continuously for 1 week, with the application of additional ATP and DNase every 24 h (30 units per day) according to the manufacturer’s protocol (Plasmid-Safe ATP-dependent DNase, Epicentre). EccDNA-enriched samples were used as templates for phi29 polymerase amplification reactions (REPLI-g Midi Kit). EccDNA was amplified at 30 °C for 2 days (46–48 h). Phi29-amplified DNA was sheared by sonication (Diagenode Bioruptor, Belgium), and the fragmented DNA was subjected to library preparation with NEBNext^®^ Ultra II DNA Library Prep Kit for Illumina (New England Biolabs, Ipswich, MA, USA). Sequencing was performed on an Illumina NovaSeq 6000 with 150 bp paired-end mode according to the manufacturer’s instructions.

### 2.3. Circular DNA Identification and Visualization

We used the GRCh38 human genome as the alignment reference. Reads were aligned using BWA-MEM (v0.7.17) [13] with the –q option, leaving the rest of the parameters on their default settings. We then used Circle-Map software (v1.1.4) [14] to detect eccDNA. EccDNA copies were identified by raw soft-clipped read counts of the breaking point. An R package ChIPseeker (v1.26.2) [15] was used to annotate the eccDNAs and plot the distribution of eccDNAs in the promoter region. All eccDNA intervals were plotted into a genome map using R packages karyoploteR (v1.16.0) [16]. The cytogenetic band enrichment analysis of eccDNA was performed using R packages clusterProfiler (v3.18.1) [17].

### 2.4. RNA Sequencing (RNA-seq) and Transcriptome Data Analyses

RNA sequencing was performed by GENEWIZ (Suzhou, China) using the Illumina system. Paired-end libraries were sequenced by an Illumina HiSeq X Ten (2 × 150-nucleotide read length), with a sequence coverage of 46 million paired reads. For the tumor and adjacent non-tumor samples, RNA-seq yielded an average of 47 million and 45 million high-quality reads, respectively (Figure S1A). We used Salmon (v1.7.0) [18] for quantifying transcript abundance from RNA-seq reads. Raw transcript counts per sample were used to perform differential expressed gene (DEG) analysis comparing groups using the R package DESeq2 (v1.30.1). Low-expression genes across samples were removed and normalized using the estimateSizeFactors function. Pathway analysis was conducted with GSEA software (v4.1.0).

### 2.5. Validation of eccDNA

PCR primers were designed in Primer3web (version 4.0) and devised to yield products across junctions of a circular DNA structure (Supplementary Table S2). Each 50 μL of the PCR reaction typically included a 120 ng template, 200–320 nM primer, dNTP, buffer, and polymerase, and PCR was for 35 cycles in a PCR cycler under standard PCR conditions. Size-separation of PCR products on agarose (~1.5%) gel electrophoresis and Sanger sequencing of PCR products confirmed the circular structure of the detected eccDNA.

### 2.6. Statistical Analysis

The correlation between the ratio of genes/million bases (Mb) and in any of the chromosomes was performed by linear regression analysis. The data from the two groups were compared by a Wilcoxon rank-sum test. *p* < 0.05 was considered statistically significant. All statistical analyses were performed using R (4.0.5).

## 3. Results

### 3.1. Genome-Wide eccDNAs Profiling by Circle-seq in Paired HCC and Adjacent Tissue Samples

**Figure 1:**
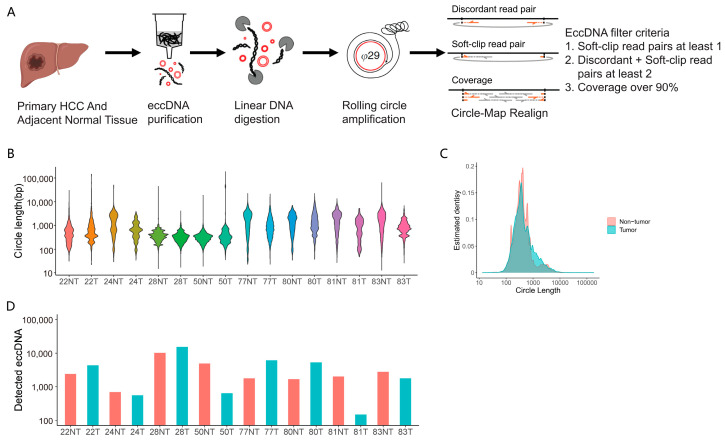
Genome-wide detection and analysis of eccDNAs profiles by Circle-seq in paired HCC and adjacent tissue samples. (**A**). Circle-Seq procedure. From primary HCC and adjacent non-tumor tissue cells, eccDNA was purified using the following procedure: (1) column purification for circular DNA; (2) digestion of remaining linear DNA by plasmid-safe ATP-dependent DNase; and (3) rolling circle amplification by ϕ29 DNA polymerase and subsequent high-throughput DNA sequencing. Detection of eccDNAs based on structural-read variants (soft-clipped reads + discordant reads ≥ 2 and soft-clipped reads ≥ 1) and coverage (90%). (**B**). Length distribution of eccDNA in the eight paired HCC and adjacent tissue samples. (**C**). Size distribution of detected eccDNAs in the eight paired HCC and adjacent tissue samples. (**D**). Unique eccDNA types in the indicated tissue samples.

To investigate eccDNA expression in HCC, we applied the Circle-Seq method to detect eccDNAs on a genomic scale in eight matched HCC and adjacent non-tumor tissue samples [6,19]. Purification, enrichment, and detection of eccDNAs in the matched samples were performed in four steps (Figure 1A): (1) DNA isolation by column separation; (2) removal of remaining linear DNA by exonuclease; (3) rolling-circle amplification; and (4) sequencing and mapping of paired-end reads to the human genome to identify structural variation resulting from DNA circularization [14]. Each detected circular DNA structure was supported by a read coverage > 90% and a minimum of two independent structural-read variants including at least one split-read that identified the chromosomal breakpoint coordinates that were joined on the eccDNA. The length of eccDNAs ranged from 13 to 63,569 bp and from 17 to 182,363 bp in adjacent non-tumor tissue and tumor tissue, respectively (Figure 1B). Of note, most of the eccDNAs were smaller than 1 kb, with mean sizes of 674 bp (adjacent non-tumor tissue) and 813 bp (tumor) (Figure 1C), and no large ecDNA was observed in the enrolled samples. In total, we detected 60,423 types (34,049 in the tumor tissue and 26,374 in the adjacent non-tumor tissue) of eccDNAs in eight paired HCC samples. The amount of eccDNA types detected between the HCC and adjacent tissue samples was generally similar (Figure 1D). However, there were few eccDNA types that were exactly the same among different samples. Even among the non-tumor tissue samples included in this study, there was rarely a complete match of eccDNA: only 16 types of eccDNAs were identified in at least 2 or more tissues in the 8 adjacent tissues. Additionally, there were 21 types of eccDNAs detected in at least 2 or more HCC tissues, distributed across different chromosomes (Tables S3 and S4). In addition, the most abundant circular DNA detected in each tissue sample is shown in Table S5. These results suggested that there was no statistical difference in length and chromosome distribution of the eccDNA pattern between the HCC and adjacent non-tumor. Although the functional role of the eccDNAs remains uncertain, it is clear that the expression of eccDNA in the tumor as well as the adjacent non-tumor tissues has an overall high heterogeneity.

### 3.2. Genomic Distribution of eccDNAs in the HCC and Adjacent Tissue Samples

**Figure 2:**
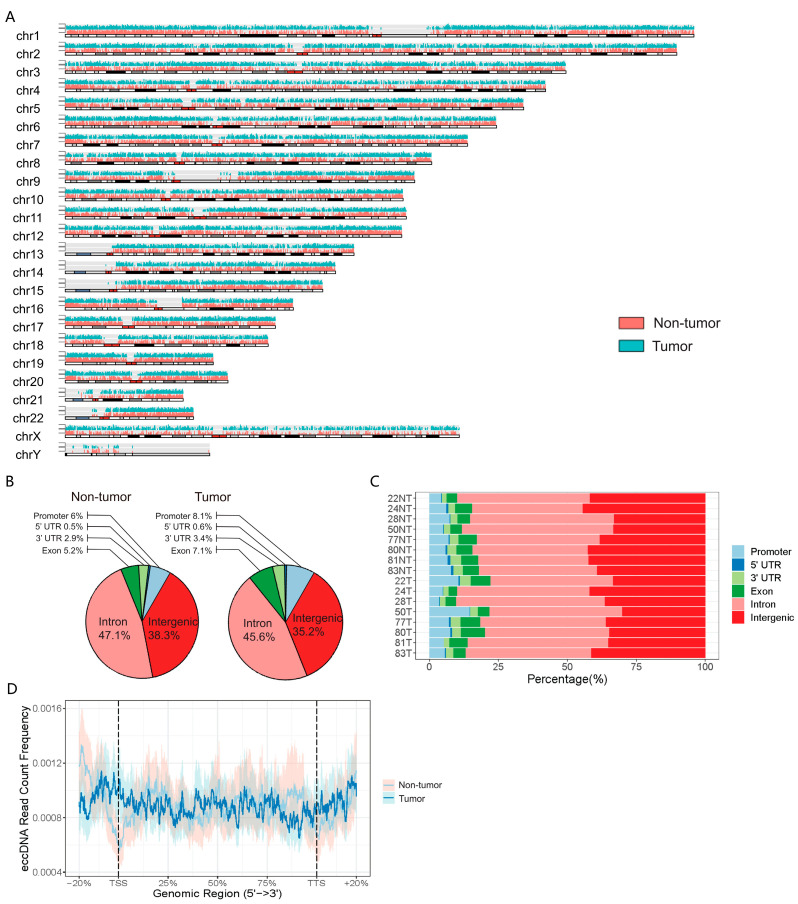
Genomic distribution of eccDNAs in the paired HCC and adjacent non-tumor tissues. (**A**). Chromosome ideogram with genome-wide somatic eccDNA density as inferred from liver tumor tissue compared to adjacent non-tumor tissue (Line height indicated the copies of eccDNA in the corresponding genome locus). (**B**). Percentage of the eccDNA types that were distributed in different genomic regions as indicated. (**C**). Genomic region distribution of eccDNAs in the indicated tissue samples. (**D**). The density of the eccDNAs mapped to the protein coding gene region. TSS, transcription start site. TTS, transcription termination site.

To provide further insight into the distribution of eccDNAs, we mapped all the eccDNAs into the genome (Figure 2A). Although genome mapping of all eccDNAs revealed that eccDNAs were widespread across the entire genome, further analysis at the genomic region scale suggested that eccDNAs were significantly enriched in genic regions rather than the intergenic region (Figure 2B,C). Due to the relatively short length, most of these eccDNAs only included fractions of genes. There was no significant difference in the distribution of eccDNAs at the gene scale. The eccDNAs were distributed evenly over the promoter, exon, intron, and et al. regions of the gene (Figure 2D).

**Figure 3:**
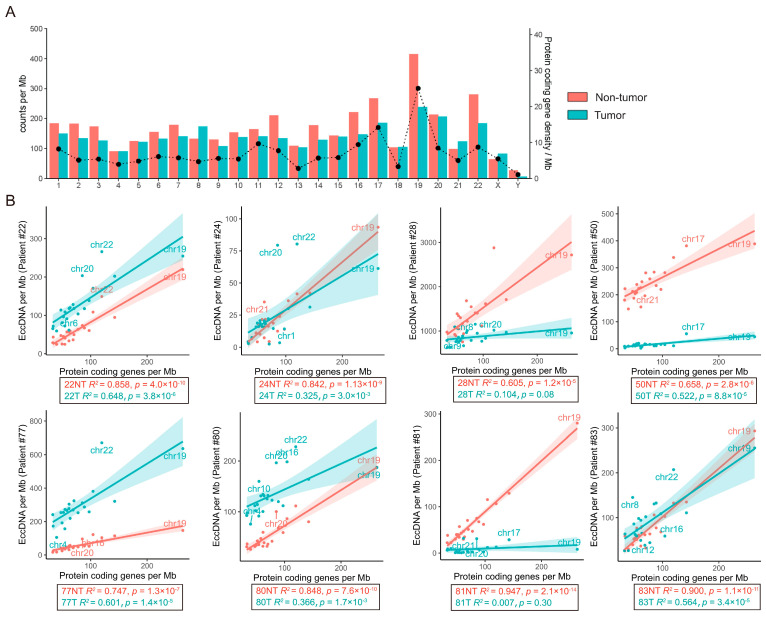
Distribution of eccDNAs along chromosomes was significantly correlated with protein coding gene density in adjacent tissue, but less correlated in tumor tissue. (**A**). eccDNA frequency relative to chromosome and coding gene density at the chromosome level (right y-axis: black line indicated the average coding gene density in all 8 paired samples, including tumor and non-tumor tissue). (**B**). The average protein coding gene density per Mb for each chromosome versus the average percentage of total eccDNAs per Mb on the chromosome. Chr19 (with the highest protein coding gene density) and the outliers were marked.

Previous studies have shown that the most frequent gains in chromosomes 1q and 8q as well as losses in chromosomes 4q, 8p, 16p, 16q, and 17p occur in HCC [20]. Thus, we further analyzed the genomic distribution of eccDNAs on different chromosomes. We found that the abundance of eccDNAs in the adjacent non-tumor tissue was slightly higher than in tumor tissue in all 24 (22 + X + Y) chromosomes. The gene-rich chromosome 19 contributed to a higher average frequency of eccDNAs per Mb than other chromosomes in the adjacent non-tumor tissue, while the gene-poor chromosome Y contributed a lower average frequency of eccDNAs per Mb than other chromosomes in both adjacent non-tumor tissue and tumor tissue (Figure 3A). A significant positive correlation between protein coding gene density and eccDNAs density was observed in all adjacent tissues (Figure 3B). Interestingly, this correlation was less significant in the corresponding tumor tissues. Particularly, among the outliers in linear regression, the number of chr22-derived eccDNAs was higher in five of the eight tumor tissues (Figure 3B), indicating a potential association between eccDNA and chr22 in HCC.

### 3.3. Transcriptomic Profiling of the Paired HCC and Adjacent Tissue Samples

**Figure 4:**
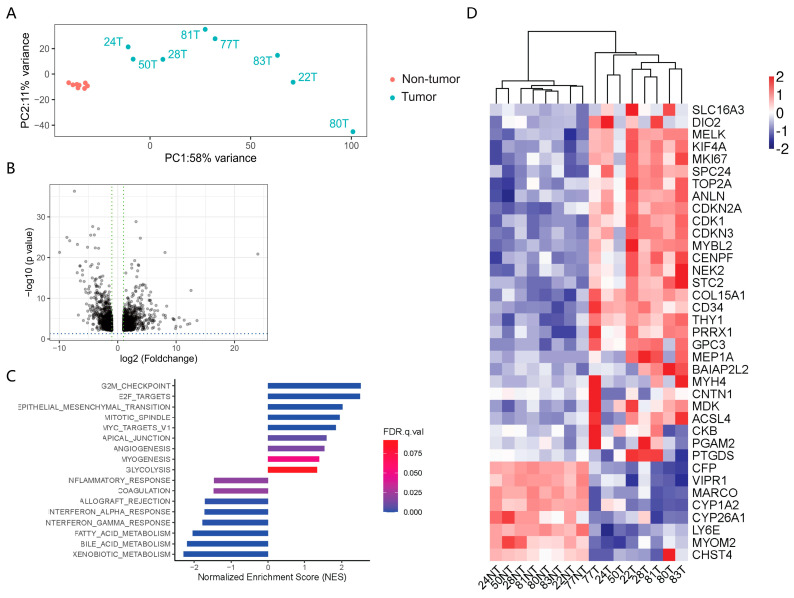
Transcriptomic profiling of the paired HCC and adjacent tissue samples. (**A**). PCA plot showing the clustering of HCC tissue and the adjacent non-tumor tissue. The variance was present between HCC tissue and adjacent non-tumor tissue on PC1. (**B**). Volcano plots of gene expression changes (HCC tissues vs. adjacent non-tumor tissues). *p*-values were estimated using the Benjamini–Hochberg method. (**C**). Pathway enrichment analysis based on the DEGs. (**D**). Heatmap of the top DEGs identified based on the eight paired samples.

EccDNAs are too small to contain protein-coding genes, but long enough to code for regulatory short RNAs or fragments of genes [7]; the expression of eccDNA could also be a byproduct of dysregulated gene expression from those unstable DNA regions [21]. It is thus important to understand the potential association between the eccDNAs profile and the transcriptomic profile. We performed RNA-seq on the eight paired HCC samples. Using principal components analysis (PCA), we found that the RNA expression profile showed a significant difference between tumor and adjacent tissue samples (Figure 4A). DEG analysis identified 1779 DEGs in the tumor tissue versus the adjacent non-tumor tissue (fold change > 2, adjusted *p* < 0.05; Figure 4B and Figure S1B). To better understand the processes in which these DEGs participate, we conducted a biological process gene set enrichment pathway analysis. Compared to the adjacent tissue samples, the HCC tissues showed significant upregulation of proliferation-related pathways, including E2F_targets and a G2M_CHECKPOINT, as well as a significant downregulation of its metabolic function, including BILE_ACID_METABOLISM and XENOBIOTIC_METABOLISM (Figure 4C,D), exhibiting patterns of gene expression in the dedifferentiation cell state.

**Figure 5:**
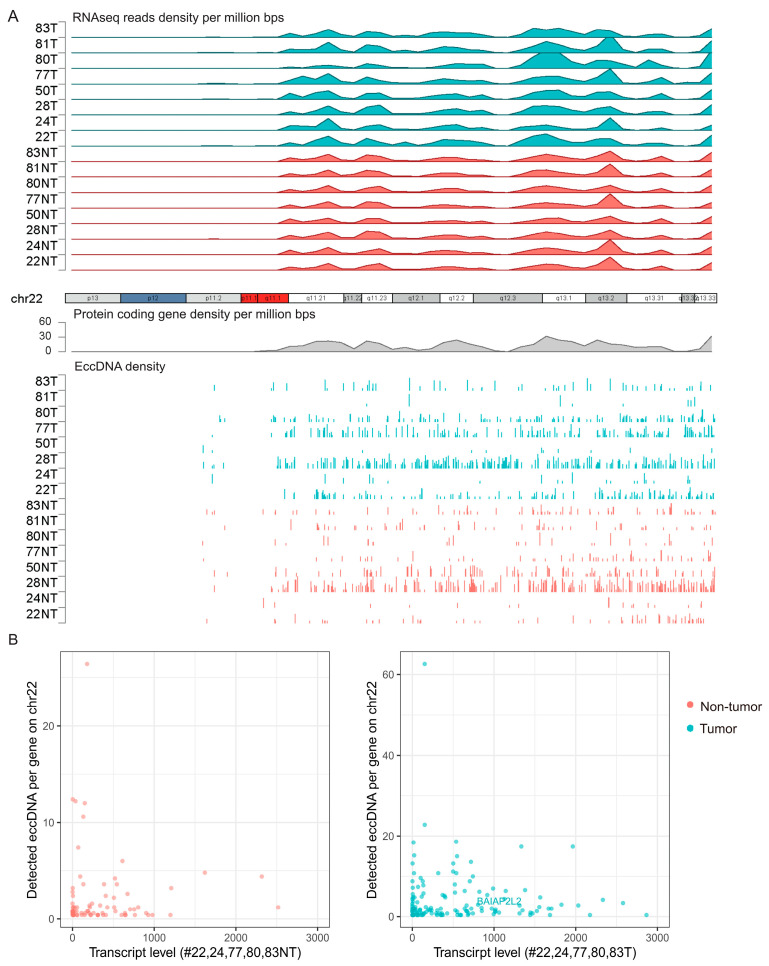
Association between eccDNA frequency and the RNA expression level. (**A**). RNAseq reads (**upper** panel), protein coding genes (**middle** panel) were grouped into bins of 1-Mb step-wise across chromosome 22. EccDNAs clustering patterns (**lower** panel) were similar to those of RNAseq reads and protein-coding gene patterns across all samples. (**B**). Relations of the eccDNA counts and the average transcription level of the five indicated (#22, 24, 77, 80, 83) samples.

EccDNA density and RNA expression levels are generally correlated in both HCC and adjacent liver tissues. The eccDNA density peaks were generally related to the RNA expression and protein-coding genes’ density peaks. The distribution patterns of the detected eccDNAs in the tumor and adjacent tissue on the chromosome level are shown in Figure 5A and Figure S2. We further measured the global mRNA level in all samples to examine whether the higher transcribed RNA levels correlated to the higher eccDNA density. Although some of the highly expressed genes gave rise to corresponding eccDNAs, no statistical correlation between the numbers of eccDNAs per gene and the transcript level was observed in both tumor and non-tumor tissues (Figure S3). As for the five HCC samples with chr22 outliers (Figure 3B), more chr22-derived eccDNA counts were detected in the tumor tissues compared to those of the adjacent non-tumor tissues (Figure 5B), supporting a relation of chr22 to the eccDNA expression in HCC.

### 3.4. Cross-Analysis of the Circle-seq Data with the Transcriptomic Data

**Figure 6:**
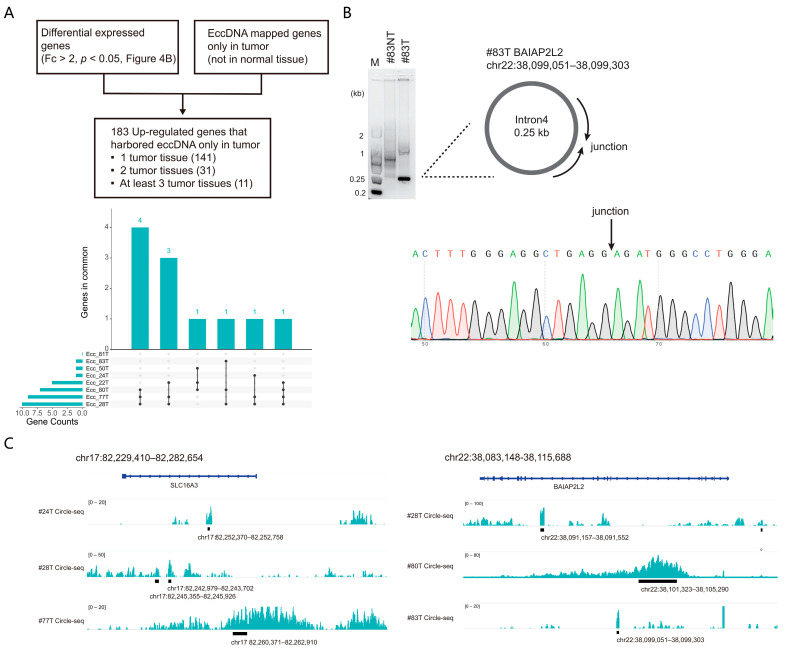
Combined analysis of RNA-seq and Circle-seq data. (**A**). Flow chart of the potential functional eccDNA analysis strategy (the **upper** panel), genes that harbored eccDNA in at least three tumor samples were indicated (the **lower** panel). (**B**). Validations for the expression of the eccDNA related to BAIAP2L2 detected in #83T, using PCR followed by gel electrophoresis and Sanger sequencing. (**C**). Genome track with paired-end reads from Circle-seq meeting the standards described above. The distribution of eccDNA reads related to the two indicated genes including SLC16A3 and BAIAP2L2 in #24T, 28T and 77T are shown.

To analyze the association between the eccDNA expression and dysregulated gene expression, we next took the intersection of the DEGs in RNA-seq and eccDNA-mapped genes. We defined the eccDNA-mapped gene as the entire eccDNA mapped to the region ranging from the start codon to the stop codon of a gene. Among the 183 up-regulated genes that related to eccDNAs exclusively detected in the tumor tissue, 11 genes harbored eccDNAs in at least three of the eight enrolled patients (Figure 6A and Table S6). Sample #83T, as an example, was subjected to validate the eccDNA expression in the BAIAP2L2 gene region by PCR and Sanger sequencing. Junctional sites were exactly the same as the eccDNAs detected by Circle-seq (Figure 6B), which proved the reliability of the Circle-seq methods.

We next ask whether the genes related to the tumor tissue-derived eccDNA associate with the survival rates in HCC. Public patient data cohorts were thus used to examine the potential role of the dysregulated genes in HCC. As a result, we found that 2 out of the 11 genes harboring eccDNAs, SLC16A3 and BAIAP2L2 showed higher expression levels and were associated with lower survival rates in HCC-related public data cohorts (Figure S4). The mapped Circle-seq reads supporting eccDNA distribution in the SLC16A3 and BAIAP2L2 genomic region are shown (Figure 6C); however, it remains unclear whether the expression of these eccDNAs plays a functional role in gene dysregulation.

## 4. Discussion

Recent studies have reported the presence of eccDNAs in most cancer types, but there have been few studies examining the profile of eccDNAs in HCC. Here, we extracted and sequenced eccDNAs in eight paired non-tumor and HCC samples. High-throughput sequencing indicated the existence and variety of eccDNAs in the paired HCC samples. Five of the eight HCC tissues showed significantly higher amounts of chr22-derived eccDNA expression compared to the non-tumor tissue. Moreover, cross-analysis of the transcriptomic and the Circle-seq data revealed that two genes, SLC16A3 and BAIAP2L2, which were highly expressed in tumor tissues, were related to eccDNAs in the tumor sample and were associated with the survival rate in HCC patient cohorts. These results provided evidence for the expression pattern of eccDNAs as well as its correlation to transcriptome dysregulation in HCC.

The diversity of eccDNAs identified in the present study supported the theory that any part of the human genome may contribute to the production of eccDNAs [6,22]. In addition, our results implied that some parts of the genome have significantly higher or lower frequencies of eccDNAs. The frequency of eccDNAs is associated with the coding gene density, which echoes previous findings in human and yeast [19,23]. For instance, the gene-rich chromosome 19 had a higher frequency of eccDNAs per Mb than other chromosomes, while the gene-poor chromosome Y had a relatively low frequency of eccDNAs per Mb. Coding genes also showed a significantly positive correlation with eccDNA copies compared to non-coding genes. One hypothesis has been suggested that transcription causes DNA damage via R-loops formed by a DNA-RNA hybrid, exposing the single-stranded DNA; R-loops form naturally during transcription but may damage DNA integrity [24]. Thus, we inferred that the accessibility of the genome may influence the eccDNA production, which may involve DNA double-strand breakage and recombination due to frequent transcription [25,26].

The mechanism of eccDNA-mediated gene regulation has been a research focus [27]. Accumulating evidence has shown that eccDNAs enhance intercellular genetic heterogeneity in tumors, especially amplification of oncogenes and drug-resistant genes [28,29,30]. In agreement with a previous study [31], we detected abundant eccDNAs (microDNAs), with most between 200 and 400 bp and less than 1 kb. They were too small to carry functional genes. A previous study has reported that artificial microDNA molecules mimicking a known microDNA sequence express functional microRNA and novel si-like RNA [10]. However, we did not detect such transcripts arising from eccDNAs in the RNA-seq analysis. Notably, we found a significant positive correlation between protein coding gene density and eccDNAs density on the chromosome scale, but this correlation was much weaker in the corresponding tumor tissues. In particular, the number of chr22-derived eccDNAs was higher in five of the eight enrolled HCC tissues, indicating a potential association between eccDNA and chr22. Interestingly, early studies have indicated that gene-rich chromosomes, such as chromosomes 19 and 22, tend to replicate early, while gene-poor chromosomes, such as 18 and 21, replicate late in cancer cell lines [32]. Loss of heterozygosity on chr22q in HCC could be a signal for malignant transformation and contribute to the HCC progression [33]. A recent paper suggested that a series of eccDNAs carrying the microRNA-17-92 cluster were identified in human HCC and the authors suggested that it could play an oncogenic role in tumor progression. However, several eccDNAs containing intact miRNAs and lncRNAs were detected as described in Table S7, but miRNA-17-92 clusters were not observed in all the eight samples included in this study. This could be due to the extremely high heterogeneity of eccDNA expression and the fact that samples included in the previous study were limited [34]. Our results suggested that the expression of eccDNAs is closely associated with gene expression. EccDNAs originating from transcriptionally active regions are more abundant. Moreover, at the level of each tissue and each cell, the expression of eccDNA types could display high heterogeneity. Identifying shared functional eccDNAs among different patients is challenging; therefore, it is possible that, in practice, different patients may have different functional eccDNAs. Thus, the present study serves as an important supplement to the previous findings and further deepens our understanding of the eccDNA expression in HCC.

Recent studies suggest that eccDNAs may be critical for the identification and prognosis of tumor development [35]. Thus, we further identified the differences in eccDNA expression and its correlation with the dysregulated genes between matched non-tumor tissue and tumor tissue. By analyzing the dysregulated genes that harbor eccDNAs exclusively detected in HCC tissue, we identified SLC16A3 and BAIAP2L2 and suggested their possible role in HCC. SLC16A3 is a member of the solute carrier family and may be responsible for the export of lactate derived from glycolysis, which was further identified as a potential prognostic biomarker associated with intrahepatic cholangiocarcinoma [36]. BAIAP2L2 is an epithelial-specific BAR domain protein involved in multiple cancers, including gastric cancer, osteosarcoma, and prostate cancer [37,38,39]. The expression level of BAIAP2L2 has been shown to be a biomarker and potential therapeutic target for lung cancer [40]. The dysregulation of the two genes could be attributed to the change in proliferation and migration; yet, their exact relationship with eccDNAs regarding cause and effect remains to be investigated. Notably, both SCL16A3 and BAIAP2L2 genes are located on chr 17, but not chr 22. We speculate that although HCC tissues showed a significantly higher amount of eccDNA expression derived from chr 22, it is not necessary for dysregulated genes associated with HCC development and harbored eccDNA to originate from chr 22. In addition to the transcriptome, the proteomic dysregulation and its relation to eccDNAs can also be considered. Further studies based on larger cohorts and functional models will help to ascertain the origin and biological role of eccDNAs in HCC progression. However, due to the extensive variety of the eccDNAs and the complexity of dysregulated pathways involved in HCC, it is technically challenging to clarify the causative role of various eccDNA molecules.

## 5. Conclusions

These results indicate the existence and significant heterogeneity of eccDNAs in HCC and adjacent liver tissues. Furthermore, there is a considerable difference in eccDNA expression between HCC and adjacent non-tumor tissues at the chromosomal level, which is associated with the transcriptome profile. These findings have deepened our understanding of the eccDNA expression patterns in HCC and its association with dysregulated gene profiles.

## Data Availability

All sequencing data were deposited in the Sequence Read Archive (SRA) under accession code number PRJCA020667.

## Supplementary Materials

The following supporting information can be downloaded at: https://www.mdpi.com/article/10.3390/cancers15225309/s1, Figure S1: The heatmap of significantly differential expressed genes in HCC tissue and the adjacent normal tissue; Figure S2: Protein coding genes (upper panel) were grouped into bins of 1-Mb step-wise across chromosome 13, 14,15; Figure S3. The relation analysis of the eccDNA counts per gene and the average transcription level of the eight HCC and adjacent tissue samples; Figure S4. Two genes related to eccDNA showed higher expression levels and are associated with lower survival rates in public HCC cohorts; Table S1: Clinical annotation of patients analyzed with Circle-seq and RNA-seq; Table S2: Primers used for PCR and Sanger DNA sequencing; Table S3: All eccDNAs identified in at least two or more tissues in the eight adjacent tissues; Table S4: All eccDNAs identified in at least two or more tissues in the eight HCC tissues; Table S5: The most abundant circular DNA detected in each tissue sample; Table S6: Genes that harbored eccDNA in at least three tumor samples; Table S7: EccDNAs containing lncRNAs and miRNAs detected in the samples.

## Abbreviations

eccDNA	extrachromosomal circular DNA
ecDNA	extrachromosomal DNA
HCC	hepatocellular carcinoma
Mb	million bases
PCA	principal components analysis
DEGs	differentially expressed genes
BAIAP2L2	BAR/IMD domain containing adaptor protein 2 like 2
SLC16A3	Solute carrier family 16 member 3
SMD	standardized mean difference
