# Supplementary material for: Genome-Wide Extrachromosomal Circular DNA Profiling of Paired Hepatocellular Carcinoma and Adjacent Liver Tissues
Source: Cancers (Basel). 2023 Nov 7;15(22):5309. doi: 10.3390/cancers15225309 (PMC10670553; doi:10.3390/cancers15225309)
Supplement: Supplementary file 1 [file cancers-15-05309-s001.zip › cancers-2623888-supplementary.pdf]

Supplementary Materials for

Genome-wide extrachromosomal circular DNA profiling of paired hepatocellular carcinoma and adjacent liver tissues

Jianyu Ye [1 #], Peixin Huang [2,3 #], Kewei Ma [1], Zixin Zhao [1], Ting Hua [1], Wenjing Zai [1], Jieliang Chen [1 *] and Xiutao Fu [2,4,5 *]

[1] Key Laboratory of Medical Molecular Virology (MOE & NHC), Research Unit of Cure of Chronic Hepatitis B Virus Infection (CAMS), School of Basic Medical Sciences, Shanghai Medical College Fudan University, Shanghai 200032, China
[2] Liver Cancer Institute, Fudan University, Shanghai 200032, China
[3] Department of Hepatic Oncology, Zhongshan Hospital, Fudan University, Shanghai 200032, China
[4] Department of Liver Surgery and Transplantation, Zhongshan Hospital, Fudan University Shanghai 200032, China
[5] Key Laboratory of Carcinogenesis and Cancer Invasion (Ministry of Education), Shanghai 200032, China
# These authors contributed equally to this work.
* Correspondence to: jieliangchen@fudan.edu.cn (J. C.); fu.xiutao@zs-hospital.sh.cn (X. F.)

Supplementary S1: R code for the main figures

Figure 1B
```
ggplot(v3,mapping = aes(x = group,y=`Circle length(bp)`,fill=group),)+
  geom_violin()+
  theme_classic()+
  theme(text = element_text(size=16,family = "sans"))+
  scale_y_log10(breaks = c(10, 100, 1000,10000,100000),
              label = c("10", "100", "1000","10000","100000"))
```

Figrue 1C
```
ggplot(NT,aes(x=log10(`Circle length (bp)`),color=group,fill=group))+
  geom_density(alpha=0.5)+
  theme_classic()+
  labs( y= "Estimated dentisy",x="Circle Length", size=10)+
  theme(text = element_text(family = "sans",
                       size=16))+
  scale_x_continuous(breaks = c(1,2,3,4,5),
                  labels = c("10",'100','1000','10000','100000'))+
  scale_y_continuous(breaks = c(0,0.5,1,1.5,2),
                  labels = c("0",'0.05','0.1','0.15','0.2'))
```

Figure 1D
```
ggplot(Ecclist_byindividual)+
  geom_bar(aes(x=sample_list,y=copies,fill=group),width = 0.5,stat = 'identity')+
  theme_classic()+
  theme(text = element_text(size=16,family = "sans"))+
  scale_x_discrete(labels = c("copies of eccDNA1" = "Nitrogen rate1"))+
  theme(text = element_text(size=16,family = "sans"))+
  scale_y_log10(breaks = c(100,1000,10000,100000),
              labels = c('100','1000','10000','100000'))+
  coord_cartesian(ylim = c(100,100000 ))+
  ylab("Detected eccDNA")+
  xlab("")
```

Figure 2A
```
plot.params <- getDefaultPlotParams(plot.type=2)
plot.params$ideogramheight <- 80
kp <- plotKaryotype(genome="hg38", plot.type=2,plot.params = plot.params)
kpDataBackground(kp, data.panel = 1, r0=0.1, r1=0.7)
kpDataBackground(kp, data.panel = 1, r0=0.8, r1=1.4)
kpAxis(kp, ymin=2, ymax=8, r0=0.2, r1=0.8, col="gray50", numticks=2,cex=0.5,text.col = 'white')
kpAxis(kp, ymin=2, ymax=8, r0=0.9, r1=1.5, col="gray50", numticks=2,cex=0.5,text.col = 'white')
kpBars(kp, chr=Norm_sum$chr, x0=Norm_sum$start, x1=Norm_sum$end, y1 =
Norm_sum$norm_copy_number,
      ymin=2, ymax=8, r0=0.2, r1=0.8, data.panel = 1, border="#F8766D" )
kpBars(kp, chr=Tumor_sum$chr, x0=Tumor_sum$start, x1=Tumor_sum$end, y1 =
Tumor_sum$norm_copy_number,
      col="#AADDAA", ymin=2, ymax=8, r0=0.9, r1=1.5, data.panel = 1, border="#00BFC4" )
legend(x = "bottomright", fill = c("#F8766D", "#00BFC4"), legend = c("Norm", "Tumor"),box.col="white")
```

Figure 2B
```
anno_Tumor_2 <- annotatePeak(Tumor,tssRegion = c(-500,500),
```

```
                    TxDb = TxDb.Hsapiens.UCSC.hg38.knownGene)
plotAnnoPie(anno_Tumor_2,ndigit=1)
anno_norm_2 <- annotatePeak(Norm,tssRegion = c(-500,500),
                     TxDb = TxDb.Hsapiens.UCSC.hg38.knownGene)
plotAnnoPie(anno_norm_2,ndigit=1)
```

Figure 2C

```
plotAnnoBar(list_all,ndigit=1)+
  theme(text = element_text(size=16,family = "sans"))
```

Figure 2D

```
plotPeakProf2(peak = list(Norm,Tumor), upstream = rel(0.2), downstream = rel(0.2),
          by = "gene", type = "body", nbin = 800,
          TxDb = TxDb.Hsapiens.UCSC.hg38.knownGene, weightCol = "V5",ignore_strand = T,
          ylab = "eccDNA Read Count Frequency",conf = 1)+
  theme(text = element_text(size=16,family = "sans"))
```

Figure 3A

```
ggplot(NT,aes(y=counts_per_Mb,x=V1,fill=group))+
  geom_bar(stat = 'identity', position = 'dodge')+
  theme_classic()+
  ylab("counts per Mb")+
  scale_y_continuous(expand = c(0,0),limits = c(0,500),
                sec.axis = sec_axis(~./12,
                             name = 'Protein coding gene density / Mb',
                             breaks = seq(0,40,10)))+
  geom_line(mapping = aes(x= V1,
           y= V2*12),
        linetype=3,cex=0.5,group=1)+
  geom_point(mapping = aes(x= V1,
                  y= V2*12),
         color='black',size=2)
```

Figure 4

Mainly following tutorial
https://www.bioconductor.org/packages/release/workflows/vignettes/rnaseqGene/inst/doc/rnaseqGene.R

Figure 5A

```
plot.params <- getDefaultPlotParams(plot.type=2)
plot.params$ideogramheight <- 10
kp <- plotKaryotype(genome="hg38", plot.type=2,plot.params = plot.params,chromosomes = c("chr22"))
kpAddCytobandLabels(kp, cex=0.5, force.all=TRUE)
kpPlotDensity(kp,data = gr0,window.size = 1e6,r0=0.15, r1=0,data.panel=2,ymin = 0,ymax = 60)
kpAxis(kp, data.panel = 2, ymin=0, ymax=40, r0=0.15, r1=0, col="gray50", numticks=3,cex=0.8,text.col =
'black',labels = c(0,30,60))
for (i in 1:length(sample_list)) {
  kpPlotDensity(kp,data = Eccgr[[i]],window.size = 1e6,r0=0.02+(i-1)*0.08,
r1=0.02+i*0.08,data.panel=1,ymin = 0,ymax = 100000,col=Ecc_color[i])
  kpAxis(kp, ymin=0, ymax=8, r0=0.02+(i-1)*0.08, r1=0.02+i*0.08, col="gray50",
numticks=2,cex=0.5,text.col = 'white')
  kpAddLabels(kp, sample_list_1[i], label.margin=0.01, side="left", offset=0, r0=0.02+(i-1)*0.08,
r1=0.02+i*0.08)
}
```

```
for (i in 1:length(Ecclist)) {
  Ecclist_chr20[[i]] <- Ecclist[[i]] %>%
    filter(V1=='chr22') %>%
    select(c(1:3,5)) %>%
    mutate(copy_number=log2(V5)) %>%
    mutate(norm_copy_number=copy_number) %>%
    `colnames<-`(c('chr','start','end','splite_read','copy_number','norm_number'))
  kpBars(kp, chr=Ecclist_chr20[[i]]$chr, x0=Ecclist_chr20[[i]]$start, x1=Ecclist_chr20[[i]]$end,
        y1 = Ecclist_chr20[[i]]$norm_number,
        ymin=0, ymax=8, r0=1.55-(i-1)*0.08, r1=1.55-i*0.08, data.panel = 2, border=Ecc_color[i])
  kpAxis(kp, ymin=0, ymax=8, r0=1.55-(i-1)*0.08, r1=1.55-i*0.08, col="gray50", numticks=2,cex=0.5,text.col
= 'white',data.panel = 2)
  kpAddLabels(kp, sample_list_1[i], label.margin=0.01, side="left", offset=0, r0=1.55-(i-1)*0.08, r1=1.55-
i*0.08,data.panel = 2)
}
```

Figure 6A

```
upset(Ecclist_bygene_full_over3,
      sets = c('Ecc_22T',
               'Ecc_24T',
               'Ecc_28T',
               'Ecc_50T',
               'Ecc_77T',
               'Ecc_80T',
               'Ecc_81T',
               'Ecc_83T'),
      mainbar.y.label = "Genes in common",
      mb.ratio = c(0.65, 0.35),
      sets.x.label = "Gene Counts",
      matrix.color = "gray23",
      sets.bar.color = "#00BFC4",
      main.bar.color = "#00BFC4",
      text.scale=c(2,2,2,2,1.6,2))
```

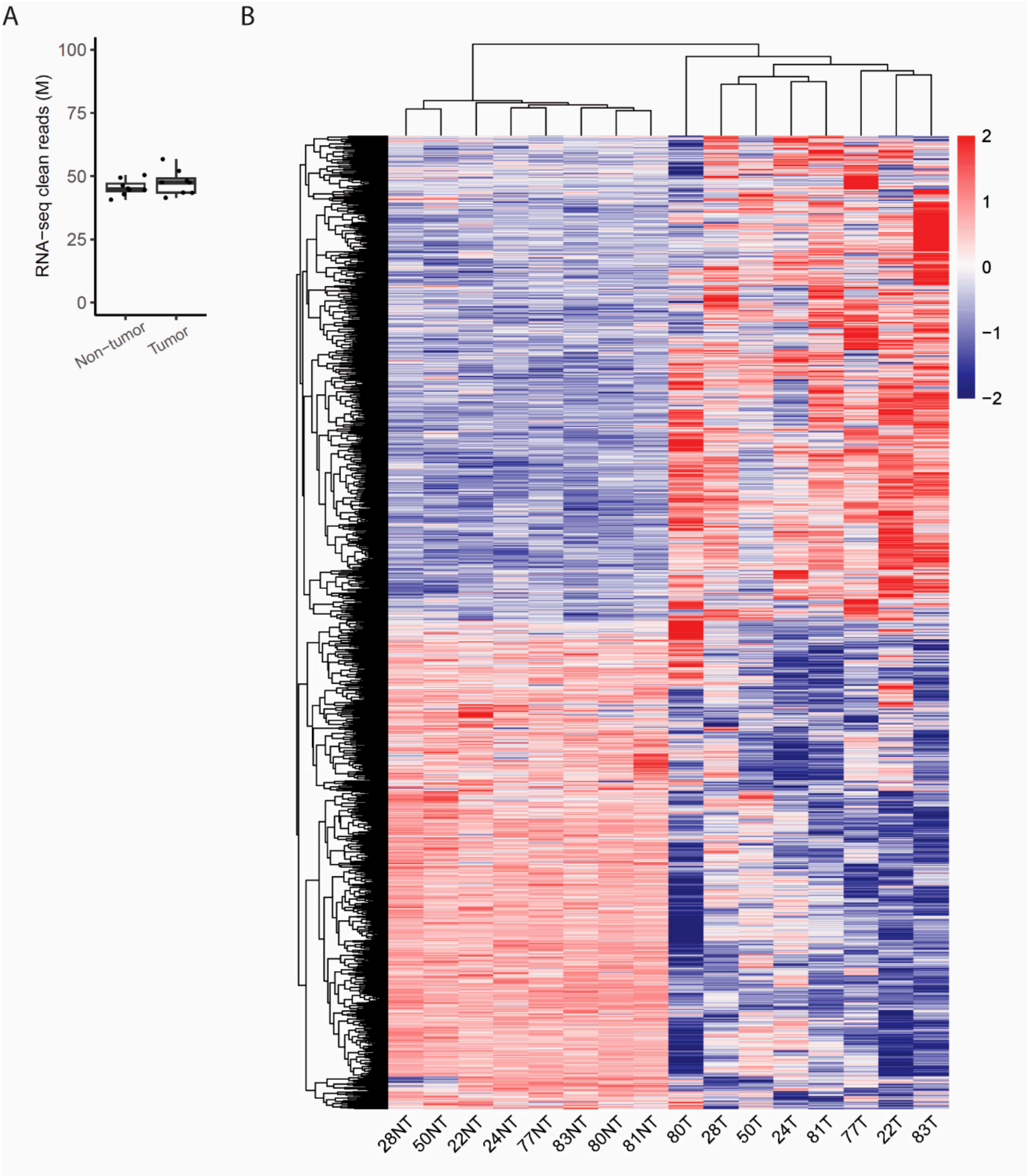

Figure S1. (A) QC passed reads in HCC and the adjacent non-tumor tissues. (B) The heatmap of significantly differential expressed genes (Fold change >2, FDR q value < 0.05, t test) in HCC tissue and the adjacent non-tumor tissue.

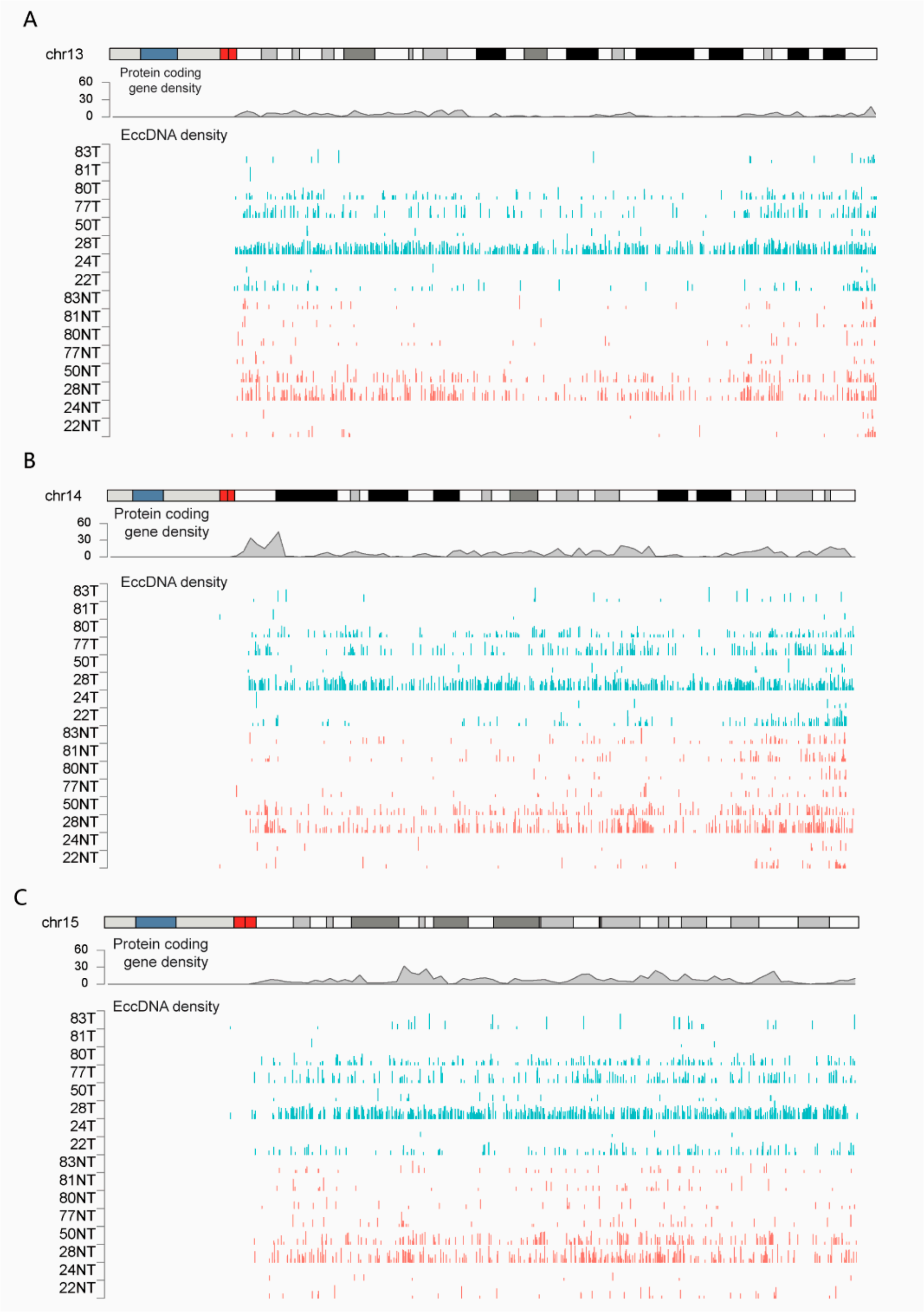

Figure S2. Protein coding genes (upper panel) were grouped into bins of 1-Mb step-wise across chromosome 13, 14,15 (A-C). EccDNAs clustering patterns (lower panel) were similar with that of protein coding gene patterns across all samples.

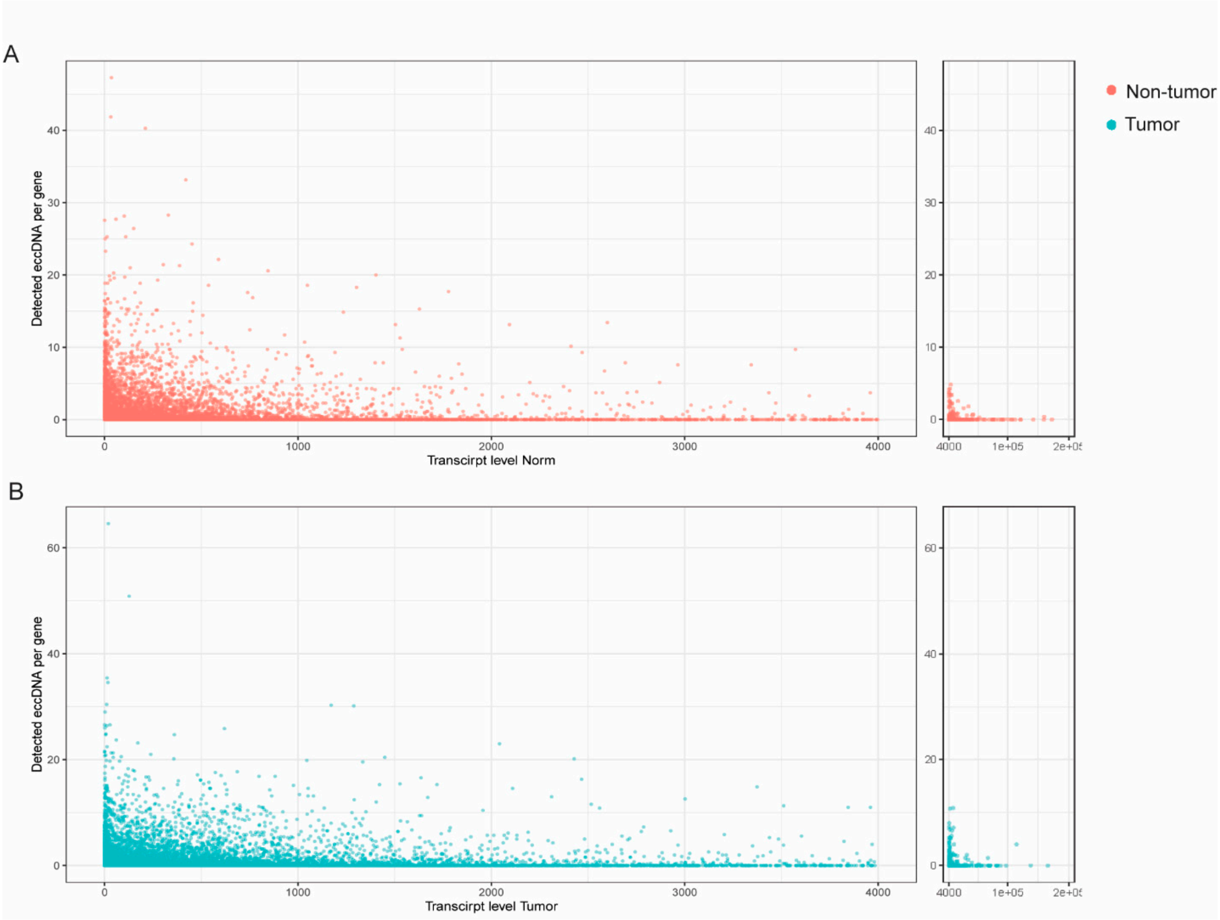

Figure S3. The relation analysis of the eccDNA counts per gene and the average transcription level of the eight HCC and adjacent tissue samples.

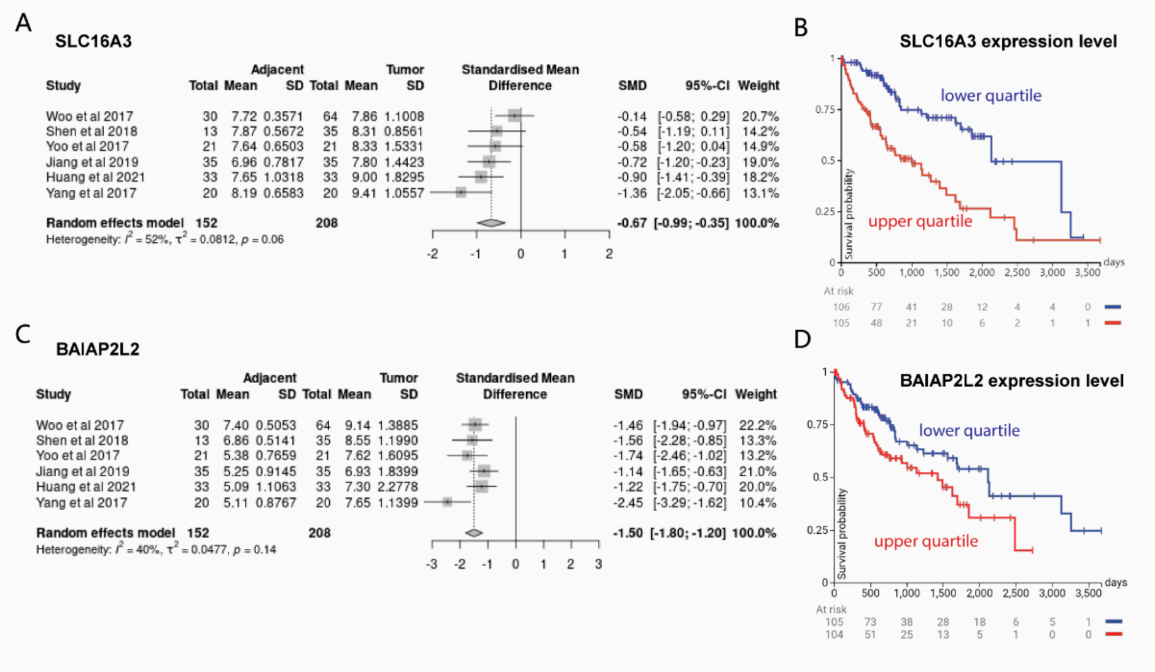

Figure S4. Two genes related to eccDNA showed higher expression levels and are associated with lower survival rates in public HCC cohorts. (A, C) Meta-analysis. Published microarray and RNA-seq data were downloaded from Gene Expression Omnibus (GEO), including accession numbers GSE76311 (1) and GSE89377 (2), as well as Bioproject, including accession numbers PRJNA310654 (3), PRJNA371753 (4), PRJNA512451 (5) and PRJNA762641 (6). We used limma (v3.46.0) to process microarray data following the standard procedure (7). After calculation of the logarithmic ratio of the gene expression level between tumor tissue and non-tumor tissue as well as its standard error for each viable study, we used the DerSimonian-Laird random effects model to perform the meta-analysis using R packages meta (v5.2-0) (8). We selected the random effects model a priori, as we expected significant study heterogeneity. Gene expression levels were combined using a random effects model. The size of each square represents the weight of that study. Microarray-derived and high-throughput sequencing gene expression levels in tumors tissues versus adjacent non-tumor liver tissues were shown. The results indicated that the standardized mean differences (SMDs) between tumor and non-tumor samples were -1.08 (95% CI -0.99 ~ -0.35) and -1.20 (95% CI -1.80 ~ -1.20) for SLC16A3 and BAIAP2L2, respectively. (B, D) Kaplan-Meier analysis comparing HCC patient survival rates with different gene expression levels in TCGA cohort. Patient survival plot was drawn based on the information summarized by the UCSC Xena (http://xena.ucsc.edu/) database. We used the GDC TCGA Liver Cancer (LIHC) project data and divided samples into two groups based on gene expression in quartiles. Two indicated genes including SLC16A3 and BAIAP2L2, showed higher expression levels and were associated with lower survival rates in HCC.

Supplementary Table S1: Clinical annotation of patients analyzed with Circle-seq and RNA-seq.

Patient ID	Age	Sex	Tissue	BCLC	CNLC	Serum HBsAg
#22	66	Male	T/NT	A	Ib	(+)
#24	70	Male	T/NT	A	Ib	(-)
#28	67	Male	T/NT	A	Ia	(+)
#50	61	Male	T/NT	A	Ib	(+)
#77	65	Female	T/NT	B	IIb	(+)
#80	56	Male	T/NT	A	Ia	(-)
#81	56	Male	T/NT	A	Ib	(+)
#83	67	Male	T/NT	A	Ib	(+)

Supplementary Table S2: Primers used for PCR and Sanger DNA sequencing

Name	Description	Sequence (5'-3')
#83T_BAIAP2L2-F	PCR of circle junction in #83_T	ACCCATCTCTGGGCCTCAGT
#83T_BAIAP2L2-R	PCR of circle junction in #83_T	GGCAACTTGTCCAAAGTCACAAAGC

Supplementary Table S3: All eccDNAs identified in at least two or more tissues in the eight adjacent tissues

chrom	start	end	length (bp)	meancov	sample
chr11	64582047	64582240	194	16.3;13.1;	50NT;83NT;
chr12	120707029	120707224	196	150.6;194.7;	22NT;80NT;
chr12	131098129	131098188	60	16.3;8.6;	28NT;50NT;
chr1	228547392	228548604	1213	14.4;63.3;	22NT;80NT;
chr12	49877510	49877621	112	4.8;60.4;	28NT;77NT;
chr1	55002911	55003057	147	146.6;65.4;	22NT;77NT;
chr15	75104289	75104508	220	29.7;18.4;	28NT;50NT;
chr17	41632758	41633278	521	65.2;77.8;	24NT;80NT;
chr17	80024304	80024653	350	72.6;46.3;40.2;	24NT;77NT;83NT;
chr20	43696476	43697013	538	13.1;117.3;	22NT;83NT;
chr3	195110258	195110895	638	23.0;215.6;	22NT;77NT;
chr9	43333269	43334072	804	196.6;470.0;357.1;75.3;	22NT;77NT;80NT;83NT;
chr9	76230379	76230727	349	7.9;9.0;	28NT;50NT;
chrY	10670737	10674059	3323	615.0;315.6;	50NT;80NT;
chrY	11048306	11048620	315	47.6;32.7;	22NT;81NT;

Supplementary Table S4: All eccDNAs identified in at least two or more tissues in the eight HCC tissues

chrom	start	end	length (bp)	meancov	sample
chr10	131176467	131177157	691	76.9;63.2;	77T;80T;
chr11	132125730	132125790	61	232.8;54.1;68.5;	22T;77T;80T;
chr12	49877510	49877621	112	28.8;47.0;	77T;80T;
chr1	30488722	30488829	108	41.3;32.7;	77T;80T;
chr1	3176048	3176298	251	14.2;26.0;	22T;77T;
chr1	55002896	55003056	161	69.9;85.2;47.2;	22T;77T;83T;
chr16	88349723	88349887	165	251.0;16.1;	22T;80T;
chr17	41632758	41633278	521	85.2;91.8;	80T;83T;
chr17	41632758	41633279	522	24.4;193.3;	24T;77T;
chr17	41632779	41633279	501	24.5;85.4;	24T;80T;
chr17	80024304	80024653	350	64.1;35.3;	77T;80T;
chr19	38750196	38750320	125	33.3;8.2;	22T;24T;
chr19	7331320	7331580	261	11.8;12.4;	22T;77T;
chr20	63913065	63913104	40	35.5;98.3;	22T;80T;
chr6	157238333	157238374	42	23.8;12.8;	22T;77T;

chr6	168270045	168270260	216	224.8;22.4;	22T;80T;
chr8	138691548	138692217	670	24.0;6.6;	77T;80T;
chr9	133254915	133255391	477	37.7;10.2;	22T;77T;
chr9	34681484	34681981	498	9.2;27.7;	28T;80T;
chrY	10670733	10674059	3327	4008.5;366.2;	24T;50T;
chrY	10670742	10674059	3318	426.2;3961.2;	80T;81T;

Supplementary Table S5: The most abundant circular DNA detected in each tissue sample. Tab separated file containing the chromosomal coordinates and coverage statistics of the detected circular DNA. Ordered from left to right, the columns contain the following information: 1,chromosome; 2, start coordinate; 3, end coordinate; 4, number of discordant read pairs; 5, number of split reads; 6, mean coverage within the detected coordinates; 7, standard deviation of the coverage within the detected coordinates; 8, read coverage ratio at the start coordinate; 9, read coverage ratio at the end coordinates; 10, fraction of reference bases not covered by sequencing reads within the detection; 11, sample type.

chrom	start	end	discordant reads	soft-clipping reads	meancov	Standard deviation	start_ratio	end_ratio	continuity	sample
chr11	66984160	66984581	11	224	403.2826603	46.9538957	0.950907778	0.974748731	0	#22T
chr20	19370979	19371626	9	230	581.3802164	87.94501291	0.991171824	0.999377482	0	#24T
chr16	46394458	46395066	14	1381	31045.99013	6985.011646	0.746902461	0.504681592	0	#28T
chr3	197489467	197490186	6	137	535.7009736	105.9972099	0.993305751	0.998638385	0	#50T
chr1	151229108	151229441	10	199	2195.912913	1220.466497	0.993774857	0.997315686	0	#77T
chr8	69572734	69573332	9	98	97.86956522	7.641182437	0.922652901	0.941573756	0	#80T
chr10	133177535	133177643	0	74	309.0555556	175.2498657	0.36929044	0.994470828	0	#81T
chr1	64223186	64224111	14	156	301.8875676	49.38600641	0.990493732	0.999975026	0	#83T
chr5	67581379	67581506	17	119	355	29.37083025	0.999972567	1	0	#22NT
chrX	49297885	49301592	26	77	363.3350418	147.8760954	0.599062381	0.99902167	0	#24NT
chr3	184826499	184826923	3674	10817	27571.92217	3101.117478	0.999981388	0.999664445	0	#28NT
chr17	2775872	2776198	22	316	488.1441718	40.16111051	0.967644048	0.98158548	0	#50NT
chr17	47066267	47066301	0	154	1047.794118	25.95786111	0.947775886	0.947775886	0	#77NT
chr19	52824741	52827890	5	135	571.269927	136.8402519	0.960918665	0.999965425	0	#80NT
chr19	45239435	45242932	6	192	379.0729196	78.52213807	0.722775412	0.65510502	0	#81NT
chr2	128454967	128455176	7	117	539.1196172	292.4763435	0.290904385	0.979230029	0	#83NT

Supplementary Table S6: Genes that harbored eccDNA in at least three tumor samples. Tab separated file containing the chromosomal coordinates and coverage statistics of the detected circular DNA. Ordered from left to right, the columns contain the following information: 1,gene name; 2.chromosome; 3, start coordinate; 4, end coordinate; 5, number of discordant read pairs; 6, number of split reads; 7, sample type.

Gene	chrom	start	end	discordant reads	soft-clipping reads	sample
LAMB3	chr1	209643261	209643707	14	20	#22T
	chr1	209637186	209637667	4	12	#28T

	chr1	209638820	209640218	16	71	#77T
NTAQ1	chr8	123435750	123435908	4	46	#22T
	chr8	123443276	123443776	0	8	#28T
	chr8	123459114	123459597	11	18	#77T
PAK3	chrX	110947727	110949064	1	2	#22T
	chrX	111106295	111106523	0	6	#28T
	chrX	111130645	111131726	0	8	#77T
	chrX	111140649	111143250	0	2	#80T
RHEX	chr1	206095741	206095922	0	2	#22T
	chr1	206074135	206074414	0	80	#28T
	chr1	206076416	206077499	0	9	#28T
	chr1	206070187	206070476	9	61	#77T
VWF	chr22	5949639	5950614	0	6	#22T
	chr22	6066228	6066586	1	8	#22T
	chr22	6056612	6056754	0	7	#50T
	chr22	6052132	6053607	2	4	#80T
SLC16A3	chr17	82252370	82252758	0	2	#22T
	chr17	82242979	82243702	0	4	#28T
	chr17	82245355	82245926	0	3	#28T
	chr17	82260371	82262910	0	2	#77T
BAIAP2L2	chr22	38091157	38091552	8	56	#28T
	chr22	38101323	38105290	0	4	#80T
	chr22	38099051	38099303	4	6	#83T
DIO2	chr14	80224449	80224797	12	17	#28T
	chr14	80308770	80309965	8	32	#77T
	chr14	80336413	80337072	0	6	#80T
GPC3	chrX	133784231	133784513	0	7	#28T
	chrX	133800579	133800769	0	4	#28T
	chrX	133634340	133638589	14	43	#77T
	chrX	133858277	133859028	24	4	#77T
	chrX	133858278	133859028	18	22	#80T
SGIP1	chr1	66700048	66700341	0	2	#28T
	chr1	66726736	66728212	0	4	#77T
	chr1	66552874	66553703	4	2	#80T
	chr1	66647568	66650015	9	19	#80T
SULF1	chr8	69556469	69556562	4	5	#28T
	chr8	69556470	69556562	0	23	#28T
	chr8	69596154	69596464	0	79	#28T
	chr8	69584303	69585219	0	2	#77T
	chr8	69572734	69573332	9	98	#80T

Supplementary Table S7: EccDNAs containing lncRNAs and miRNAs detected in the samples.

chrom	start	end	discordant reads	soft-clipping reads	Sample	Gene_start	Gene_end	Gene_information
chr1	109595947	109598302	2	2	22NT	109596225	109597781	ID=ENSG00000225113.1;gene_type=lncRNA;gene_name=RP5-1160K1.3
chr17	28360592	28360790	0	13	22NT	28360654	28360734	ID=ENSG00000284532.1;gene_type=miRNA;gene_name=MIR4723
chr2	202336371	202338639	1	3	22NT	202336739	202337200	ID=ENSG00000272966.1;gene_type=lncRNA;gene_name=RP11-686O6.1
chr8	22244913	22245377	0	4	22NT	22244962	22245043	ID=ENSG00000208037.1;gene_type=miRNA;gene_name=MIR320A
chr1	26554115	26556031	2	4	22T	26554542	26554593	ID=ENSG00000238705.1;gene_type=miRNA;gene_name=MIR1976
chr12	125149347	125172015	4	11	22T	125150058	125151394	ID=ENSG00000256814.1;gene_type=lncRNA;gene_name=RP11-158L12.5
chr12	125149347	125172015	4	11	22T	125159331	125160397	ID=ENSG00000287240.1;gene_type=lncRNA;gene_name=RP11-158L12.8
chr14	105616348	105760979	9	15	22T	105621116	105621180	ID=ENSG00000274172.1;gene_type=miRNA;gene_name=MIR8071-1
chr14	105616348	105760979	9	15	22T	105640168	105640232	ID=ENSG00000277030.1;gene_type=miRNA;gene_name=MIR8071-2
chr14	105616348	105760979	9	15	22T	105647924	105649057	ID=ENSG00000253364.2;gene_type=lncRNA;gene_name=COPDA1
chr19	41220696	41222077	2	8	22T	41221426	41222051	ID=ENSG00000269652.1;gene_type=lncRNA;gene_name=CTD-2195B23.3
chr19	10779444	10785208	0	2	22T	10780254	10780335	ID=ENSG00000265879.1;gene_type=miRNA;gene_name=MIR4748
chr19	41398633	41403844	0	2	22T	41399372	41400365	ID=ENSG00000268987.1;gene_type=lncRNA;gene_name=CTC-435M10.10
chr19	35257537	35266647	0	2	22T	35262846	35264804	ID=ENSG00000268947.1;gene_type=lncRNA;gene_name=AD000684.2
chr8	27433295	27433823	4	10	22T	27433370	27433434	ID=ENSG00000273836.1;gene_type=miRNA;gene_name=MIR6842
chr9	21995422	21997233	3	4	22T	21995482	21996013	ID=ENSG00000266446.1;gene_type=lncRNA;gene_name=RP11-149I2.4
chr19	45436126	45437170	1	2	24T	45436654	45436704	ID=ENSG00000275726.1;gene_type=miRNA;gene_name=MIR6088
chr19	45436367	45437014	0	4	24T	45436654	45436704	ID=ENSG00000275726.1;gene_type=miRNA;gene_name=MIR6088
chr14	95183293	95189156	4	15	77NT	95185117	95185854	ID=ENSG00000259143.1;gene_type=lncRNA;gene_name=CTD-2240H23.2
chr17	8317025	8319538	0	4	77NT	8318088	8318712	ID=ENSG00000226871.1;gene_type=lncRNA;gene_name=AC135178.7
chr1	174021216	174023028	2	3	77T	174022509	174022985	ID=ENSG00000224977.1;gene_type=lncRNA;gene_name=LINC02776
chr10	102432869	102437760	6	22	77T	102436512	102436584	ID=ENSG00000202569.4;gene_type=miRNA;gene_name=MIR146B
chr15	78298920	78300072	0	5	77T	78299701	78299924	ID=ENSG00000277482.1;gene_type=lncRNA;gene_name=RP11-762H8.5
chr19	53751436	53753268	0	2	77T	53752397	53752481	ID=ENSG00000207992.1;gene_type=miRNA;gene_name=MIR519A1
chr4	146407661	146409488	0	5	77T	146408583	146408688	ID=ENSG00000278438.1;gene_type=miRNA;gene_name=MIR7849
chr2	27061755	27062974	6	2	80NT	27062428	27062907	ID=ENSG00000272148.1;gene_type=lncRNA;gene_name=RP11-195B17.1
chr21	41580435	41583373	6	38	80NT	41580475	41582887	ID=ENSG00000286027.1;gene_type=lncRNA;gene_name=PCSEAT
chr7	71305736	71309221	10	24	80NT	71307672	71307770	ID=ENSG00000265878.2;gene_type=miRNA;gene_name=MIR3914-1
chr7	71305736	71309221	10	24	80NT	71307674	71307768	ID=ENSG00000283278.1;gene_type=miRNA;gene_name=MIR3914-2
chr9	40928332	40929259	8	32	80NT	40929010	40929092	ID=ENSG00000275377.1;gene_type=miRNA;gene_name=MIR1299

chr1	53265800	53272874	0	2	80T	53267935	53268601	ID=ENSG00000234578.1;gene_type=lncRNA;gene_name=RP4-784A16.1
chr10	3832337	3835925	0	2	80T	3833950	3834728	ID=ENSG00000230573.1;gene_type=lncRNA;gene_name=LINC02639
chr13	106504829	106506815	0	2	80T	106506046	106506713	ID=ENSG00000274204.1;gene_type=lncRNA;gene_name=RP11-272L14.2
chr15	98282055	98286736	0	3	80T	98282075	98285907	ID=ENSG00000259041.1;gene_type=lncRNA;gene_name=RP11-167B3.1
chr15	100887654	100890127	0	14	80T	100888472	100889106	ID=ENSG00000278456.1;gene_type=lncRNA;gene_name=RP11-66B24.9
chr17	28373141	28373578	0	16	80T	28373256	28373562	ID=ENSG00000277450.1;gene_type=lncRNA;gene_name=CTB-96E2.10
chr17	45396357	45399680	2	10	80T	45396932	45397477	ID=ENSG00000267344.1;gene_type=lncRNA;gene_name=CTB-39G8.3
chr17	59365275	59367020	0	2	80T	59366083	59366154	ID=ENSG00000263857.1;gene_type=miRNA;gene_name=MIR4729
chr18	79702231	79706460	1	6	80T	79702721	79703651	ID=ENSG00000266901.1;gene_type=lncRNA;gene_name=RP11-567M16.5
chr2	113823606	113831647	6	22	80T	113829390	113831119	ID=ENSG00000244063.1;gene_type=lncRNA;gene_name=AC024704.2
chr2	231892199	231893277	1	4	80T	231892242	231892298	ID=ENSG00000222246.1;gene_type=miRNA;gene_name=MIR1471
chr2	241844358	241846078	4	2	80T	241844380	241845036	ID=ENSG00000235151.1;gene_type=lncRNA;gene_name=AC114730.2
chr20	10997842	11002402	0	2	80T	11001304	11001763	ID=ENSG00000270777.1;gene_type=lncRNA;gene_name=RP11-103J8.2
chr22	30731362	30732037	2	16	80T	30731557	30731641	ID=ENSG00000264661.3;gene_type=miRNA;gene_name=MIR3200
chr22	48270763	48275784	8	6	80T	48274364	48274415	ID=ENSG00000266508.1;gene_type=miRNA;gene_name=MIR3201
chr3	194127063	194131864	0	4	80T	194130616	194131201	ID=ENSG00000232874.1;gene_type=lncRNA;gene_name=RP11-135A1.2
chr6	169724977	169726903	0	6	80T	169725091	169725854	ID=ENSG00000232640.1;gene_type=lncRNA;gene_name=RP1-266L20.2
chr6	42921691	42934324	0	2	80T	42928287	42929457	ID=ENSG00000287825.1;gene_type=lncRNA;gene_name=RP3-475N16.9
chr8	56220648	56226976	3	7	80T	56222688	56223173	ID=ENSG00000272343.1;gene_type=lncRNA;gene_name=RP11-140I16.3
chr1	24959521	24965572	4	2	81NT	24961345	24963097	ID=ENSG00000229162.1;gene_type=lncRNA;gene_name=RUNX3-AS1
chr1	2325727	2330894	6	14	81NT	2326201	2326693	ID=ENSG00000272161.1;gene_type=lncRNA;gene_name=RP4-713A8.1
chr11	115526569	115533475	5	32	81NT	115532322	115532953	ID=ENSG00000256281.1;gene_type=lncRNA;gene_name=RP11-136I14.2
chr12	48993774	48996423	2	2	81NT	48995150	48996334	ID=ENSG00000258283.1;gene_type=lncRNA;gene_name=RP11-386G11.3
chr17	38824473	38827671	0	2	81NT	38825838	38825892	ID=ENSG00000274054.1;gene_type=miRNA;gene_name=MIR4727
chr19	9720215	9722707	0	3	81NT	9721903	9722410	ID=ENSG00000277587.1;gene_type=lncRNA;gene_name=CTD-3116E22.8
chr20	63727249	63730493	2	2	81NT	63730072	63730377	ID=ENSG00000274501.1;gene_type=lncRNA;gene_name=RP4-583P15.16
chr20	63727250	63730511	2	4	81NT	63730072	63730377	ID=ENSG00000274501.1;gene_type=lncRNA;gene_name=RP4-583P15.16
chr5	173346158	173349727	20	31	81NT	173347455	173347536	ID=ENSG00000274994.1;gene_type=miRNA;gene_name=MIR8056
chr5	1305213	1311059	3	2	81NT	1309310	1309377	ID=ENSG00000263670.1;gene_type=miRNA;gene_name=MIR4457
chr9	128595595	128597711	7	29	81NT	128595893	128596633	ID=ENSG00000280474.1;gene_type=lncRNA;gene_name=RP11-216B9.8
chr1	162036144	162040922	12	23	83NT	162039016	162039567	ID=ENSG00000227818.1;gene_type=lncRNA;gene_name=RP11-227F8.2
chr15	94598868	94601165	0	2	83NT	94600014	94600821	ID=ENSG00000259331.1;gene_type=lncRNA;gene_name=RP11-57P19.1
chr19	18567689	18570023	0	2	83NT	18568506	18569375	ID=ENSG00000268983.1;gene_type=lncRNA;gene_name=AC005253.4
chr2	45011216	45017588	11	8	83NT	45013214	45013668	ID=ENSG00000231156.1;gene_type=lncRNA;gene_name=AC093702.1

41865555	41873239	12	19	83NT	41870633	41872054	ID=ENSG00000236545.1;gene_type=lncRNA;gene_name=AP001619.3
77697931	77698197	0	6	28NT	77698012	77698116	ID=ENSG00000266325.1;gene_type=miRNA;gene_name=MIR3665
31704618	31705437	3	86	28NT	31704728	31705260	ID=ENSG00000276867.1;gene_type=lncRNA;gene_name=CTD-2358C21.5
3960913	3961814	0	35	28NT	3961414	3961512	ID=ENSG00000283928.1;gene_type=miRNA;gene_name=MIR637
88745417	88746031	0	10	28NT	88745836	88745905	ID=ENSG00000265873.1;gene_type=miRNA;gene_name=MIR4289
155396006	155398176	8	4	28T	155396010	155396978	ID=ENSG00000227773.1;gene_type=lncRNA;gene_name=ASH1L-IT1
60478066	60478335	0	17	28T	60478111	60478181	ID=ENSG00000266140.1;gene_type=miRNA;gene_name=MIR4533
535776	535914	4	14	28T	535840	535882	ID=ENSG00000264233.1;gene_type=miRNA;gene_name=MIR4456
21470864	21471181	4	8	28T	21471058	21471146	ID=ENSG00000221783.1;gene_type=miRNA;gene_name=MIR1183
177029263	177029901	0	14	50NT	177029363	177029445	ID=ENSG00000202609.4;gene_type=miRNA;gene_name=MIR488
109833236	109833474	0	4	50T	109833348	109833436	ID=ENSG00000263510.1;gene_type=miRNA;gene_name=MIR4497

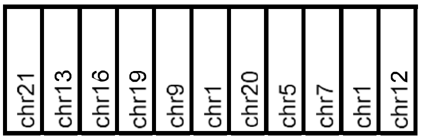
chr21
chr13
chr16
chr19
chr9
chr1
chr20
chr5
chr7
chr1
chr12
